# Effect of traditional Chinese medicine (Xiaochaihu Tang) on the expression of MMP-2 and MMP-9 in rats with endometriosis

**DOI:** 10.3892/etm.2013.1316

**Published:** 2013-09-26

**Authors:** LUYANG JIAO, XIAOFEN QI, GUANGJIAN LU, QUNMEI ZHANG, CHUNXIAO ZHANG, JIANHUI GAO

**Affiliations:** 1 Department of Laboratory, The First Affiliated Hospital of Xinxiang Medical University, Weihui, Henan 453100, P.R. China; 2 Department of Emergency, The First Affiliated Hospital of Xinxiang Medical University, Weihui, Henan 453100, P.R. China; 3 Department of Blood Transfusion, The First Affiliated Hospital of Xinxiang Medical University, Weihui, Henan 453100, P.R. China; 4 Department of Tuberculosis III, The First Affiliated Hospital of Xinxiang Medical University, Weihui, Henan 453100, P.R. China; 5 Department of Science and Technology, Xinxiang Medical University, Xinxiang, Henan 453003, P.R. China

**Keywords:** endometriosis, Xiaochaihu Tang, matrix metalloproteinase, western blotting

## Abstract

The aim of this study was to explore the effect of a traditional Chinese medicine (Xiaochaihu Tang, XCHT) on the expression of matrix metalloproteinase-2 (MMP-2) and MMP-9 in rats with endometriosis (EMs). A total of 48 specific-pathogen-free (SPF) female Sprague-Dawley (SD) rats were randomly divided into control (n=8) and EMs (n=40) groups. The EMs model was established using a surgical procedure. At 21 days, the rats with EMs were screened and divided into four subgroups (n=8): the model control, low-dose (7.5 g/kg) XCHT-treated, high-dose (15 g/kg) XCHT-treated and gestrinone-treated (0.5 mg/kg) groups. Following 21 days of treatment, the rats were sacrificed. Reverse transcription-polymerase chain reaction (RT-PCR) and western blotting were used to examine the mRNA and protein levels of MMP-2 and MMP-9 in the endometrium. The expression levels of MMP-2 and MMP-9 were significantly increased in the rats with EMs compared with those in normal rats. Moreover, XCHT was able to significantly inhibit the expression of MMP-2 and MMP-9 compared with that in the model control group. In conclusion, XCHT was able to decrease the expression of MMP-2 and MMP-9 in the ectopic endometrium. The present results may provide a potential theoretical basis for the therapy of EMs.

## Introduction

Endometriosis (EMs) is a common gynecological disease that frequently occurs in females within childbearing age, with the clinical manifestations of abdominal pain, dysmenorrhea, infertility and irregular menstruation. The disease has shown an increasing incidence in recent years, resulting in a serious impact on the health of females. EMs is a type of hormone-dependent disease. There are a number of methods used in the treatment of EMs; however, the effects are not satisfactory and the recurrence rate remains high. The pathogenesis of EMs has not yet been fully elucidated. The endometrial implantation theory (1), which has been accepted by the majority of the scientific community, proposes that the endometrium that is shed during menstruation is transported by retrograde flow with the blood through the fallopian tubes and into the abdominal cavity, where the endometrial tissue subsequently grows on the ovary and adjacent pelvic peritoneum and evolves into an ectopic endometrium. Based on this theory, a study investigated the manner of the endometrial implantation in the abdominal cavity, and revealed that the degradation and reconstruction of the extracellular matrix (ECM) (2) were the central features of endometrial implantation. Furthermore, matrix metalloproteinases (MMPs) were identified to be important in the degradation and reconstruction of the ECM.

Xiaochaihu Tang (XCHT) is a classic formulation, which is described in a ‘Treatise on Cold Pathogenic and Miscellaneous Diseases’ as having the effects of anti-inflammatory, gastric protection and regulation of the immune system (3). Previous studies have shown that XCHT exerted a good therapeutic effect on EMs in rats (3–5); however the mechanisms underlying the effect were not clear. In the present study, the mechanisms of XCHT in the treatment of EMs were investigated by observing the effect of XCHT on the expression levels of MMP-2 and MMP-9 in EMs tissues in a rat model of the disease.

## Materials and methods

### Experimental animals

Forty-eight specific-pathogen-free (SPF) female Sprague-Dawley (SD) rats, weighing 200–220 g, were provided by the Animal Laboratory Center of Zhengzhou University (Zhengzhou, China), with license no. SCXK (Henan) 2005-0001. This study was carried out in strict accordance with the recommendations in the Guide for the Care and Use of Laboratory Animals of the National Institutes of Health (8th edition, 2011). The animal use protocol was reviewed and approved by the Institutional Animal Care and Use Committee (IACUC) of Xinxiang Medical University (Xinxiang, China).

### Drugs

XCHT was obtained with reference to Zhang Zhongjing’s ‘Treatise on Cold Pathogenic and Miscellaneous Diseases’, and comprised seven crude drugs: 24 g *Bupleurum chinense*, 9 g *Scutellaria baicalensis*, 6 g ginseng root, 9 g Pinellia, 5 g licorice, 9 g ginger and 4 g jujube. These seven herbs were purchased from Henan Zhang Zhong Jing Pharmacy (Zhengzhou, China). The mixture of herbs was dipped in water for 1 h. Following this, the herbs were heated to continuously boil for 30 min, prior to the residue being decocted again for a further 30 min. The two decoctions were subsequently mixed and concentrated to 1 g/ml (crude dosage). Gestrinone capsules (2.5 mg/particle, lot no. 53110602) were purchased from Beijing Zizhu Pharmaceutical Co., Ltd. (Beijing, China).

### Animal model

Rats with a normal estrous cycle were selected using a vaginal smear method and were subcutaneously injected with stilbestrol (0.1 ml/kg) to synchronize the estrous cycle. The animal model was established according to the method described by Jones (6). Each rat was injected with 10% chloral hydrate (300 mg/kg) for anesthesia. Following routine disinfection, the abdominal cavity was opened and the uterine vessel was ligated. An incision of ~1 cm was made in the left horn of the uterus, prior to the endometrium being separated in Rockwell nutrient solution and cut into fragments of 5×5 mm. The fragment was adhered to the intimal surface of the abdominal muscles, attached to the two sides of the abdominal wall and the incision was then sutured. The remainder of the membrane was sent for pathological inspection to confirm that the tissues were endometrial. Following surgery, the rats were injected with 0.1 ml penicillin daily for five days, in order to prevent infection. The normal control group underwent sham surgery, consisting solely of the abdominal cavity being opened. Twenty-one days subsequent to the model being established, the ectopic endometrium was observed by opening the abdominal cavity under aseptic conditions. If the volume of the ectopic endometrium was observed to have increased and if a cystic capsule with transparent liquid and blood vessels was apparent, it was considered that the EMs model was successfully established. In the present experiment, out of the 40 rats that were used, 33 survived, with a success percentage of 82.5%.

### Experimental groups and administration

Thirty-two of the model rats were randomly divided into four groups, with eight rats in each group: the model control (untreated), high-dose (15 g/kg) XCHT-treated, low-dose (7.5 g/kg) XCHT-treated and gestrinone-treated (0.5 mg/kg) groups. A further eight rats subjected to a sham surgery were used as a normal control group. The rats in the normal control and untreated groups were administered an equivalent amount of normal saline, while the treatment groups were treated with the corresponding drugs. The saline and drugs were administered daily by gavage for 21 days. Following the final administration, the abdominal cavity of the rats was opened under aseptic conditions and the eutopic and ectopic endometria were separated for analysis of the mRNA and protein levels of MMP-2 and MMP-9.

### Hematoxylin and eosin (H&E) staining

Fresh tissues were fixed in 10% formalin and paraffin-embedded, prior to being cut into sections of 4 μm. The sections were successively treated with xylene, anhydrous ethanol, 90% ethanol and 70% ethanol, then dipped in distilled water for 2 min and used for H&E staining for 5–10 min. Excess dyes were washed away using tap water. The sections were subsequently gradient dehydrated in 70 and 90% ethanol for 10 min, respectively, prior to being stained with eosin for 2–3 min. The tissues were dehydrated, rendered transparent and mounted prior to being analyzed under a microscope.

### Reverse transcription-polymerase chain reaction (RT-PCR)

Total RNA was extracted from the tissues of the eutopic and ectopic endometria using TRIzol^®^ reagent (Invitrogen Life Technologies, Carlsbad, CA, USA) and reverse-transcribed into cDNA. The transcription procedure was as follows: 2 μl RNA, 3 μl oligo(dT) and 9 μl distilled water were mixed and incubated at 70°C for 5 min, prior to being removed and promptly placed on ice. Following this, 6 μl 5X buffer, 1 μl Moloney murine leukemia virus (M-MLV; Promega Corp., Madison, WI, USA), 1.5 μl deoxyribonucleotide triphosphate (dNTP) and distilled water were added up to a total volume of 30 μl. The mixture was subsequently incubated at 42°C for 60 min and then heated at 95°C for 5 min to deactivate the reverse transcriptase.

The PCR reaction system comprised 2 μl cDNA, 2.5 units Taq DNA polymerase, 5 μl 10X buffer, 1.5 mM MgCl_2_, 0.2 mM dNTP and 10 pmol/l of each primer, with distilled water added to provide a total volume of 50 μl. The primers were purchased from Shanghai Sangon Biotechnology Co. Ltd. (Shanghai, China); the sequences of the primers are shown in Table I. The PCR conditions were as follows: one cycle of 95°C for 5 min, 35 cycles of 94°C for 30 sec, Tm°C for 1 min (Tm was dependent on the primer set, see Table I) and 72°C for 40 sec, and one cycle of 72°C for 10 min. The PCR products were subsequently electrophoresed in 1.5% agarose gel, prior to being stained with ethidium bromide. The images were collected under an ultraviolet (UV) lamp and analyzed using Quantity One^®^ 1-D Analysis Software v4.6 (Bio-Rad, Hercules, CA, USA).

### Western blotting

The tissues of the eutopic and ectopic endometria were mixed using radio-immunoprecipitation assay (RIPA) lysis buffer at a volume ratio of 1:10. The lysates were homogenized using an ultrasonic homogenizer, and then maintained on ice for 10 min prior to centrifuging at 12,000 × g for 20 min. Following this, the supernatant was collected and the concentration of protein was determined using the Coomassie Brilliant Blue method. Protein samples were separated using sodium dodecyl sulfate-polyacrylamide gel electrophoresis (SDS-PAGE) and transferred to a polyvinylidene difluoride (PVDF) membrane. The membrane was then dipped in 5% skimmed milk powder for 2 h to block the nonspecific binding sites prior to incubation with the primary antibody (for MMP-2, 1:2,000; for MMP-9, 1:2,000; or for GAPDH, 1:4,000) at 4°C overnight. The MMP-2 and MMP-9 antibodies were purchased from Santa Cruz Biotechnology, Inc. (Santa Cruz, CA, USA). GAPDH and secondary antibodies were purchased from Shanghai Kangcheng Biotechnology Co. Ltd. (Shanghai, China). After rinsing in Tris-Buffered Saline and Tween 20 (TBST) three times, the membrane was incubated with the secondary antibody (1:5,000), which was labeled with horseradish peroxidase (HRP), for 4 h, and then rinsed in TBST a further three times. Images were obtained using electrochemiluminescence (ECL), prior to being analyzed using Quantity One^®^ 1-D Analysis Software v4.6.

### Statistical analysis

The data are expressed as the mean ± standard deviation and were analyzed using SPSS 12.0 statistical software (SPSS, Inc., Chicago, IL, USA). An analysis of variance (ANOVA) test was used to compare the scores of different groups. Post hoc (least significant difference, LSD) tests were performed for comparisons between groups. P<0.05 was considered to indicate a statistically significant difference.

## Results

### Histological staining of endometrial tissues

**Figure 1:**
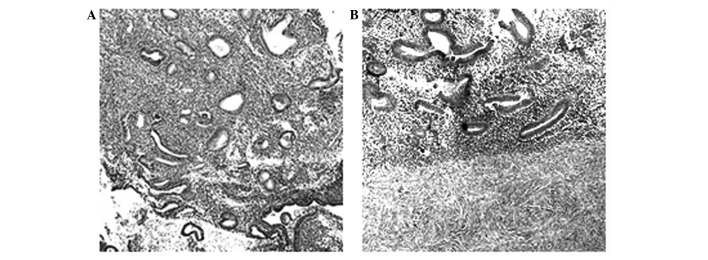
Morphology of the endometrium (hematoxylin and eosin staining; magnification, ×200). (A) Normal and (B) ectopic endometrium.

H&E staining showed that the ectopic endometrium was covered with connective tissues and that the glands and intercellular substances grew well and intensively. The intima was thick and the glandular and superficial epithelia formed a high column. Evident hyperplasia and angiopoiesis were observed, as shown in Fig. 1.

### Expression of MMP-2 and MMP-9 mRNA

**Figure 2:**
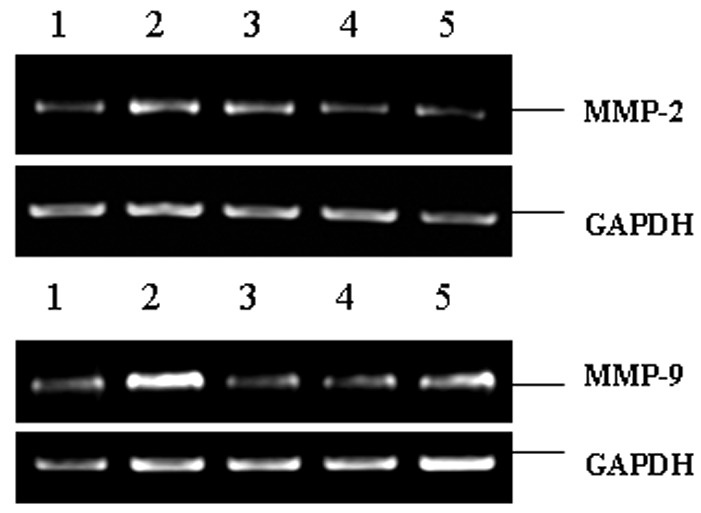
Effect of Xiaochaihu Tang (XCHT) on mRNA levels of matrix metalloproteinase (MMP)-2 and -9. GAPDH, glyceraldehyde 3-phosphate dehydrogenase. Lane 1, sham surgery; lane 2, model control; lane 3, 7.5 g/kg XCHT group; lane 4, 15 g/kg XCHT group; lane 5: gestrinone.

As shown in Fig. 2 and Table II, the expression levels of MMP-2 and MMP-9 mRNA were low in the normal endometria, but increased significantly in the ectopic endometria (P<0.01 and P<0.001, respectively). Following XCHT administration, the MMP-2 and MMP-9 mRNA levels were significantly decreased compared with those in the model group (P<0.05 and P<0.001, respectively, for the 7.5 g/kg group; P<0.01 and P<0.001, respectively, for the 15 g/kg group). These results indicate that XCHT reduced the mRNA levels of MMP-2 and MMP-9.

### Protein levels of MMP-2 and MMP-9

**Figure 3:**
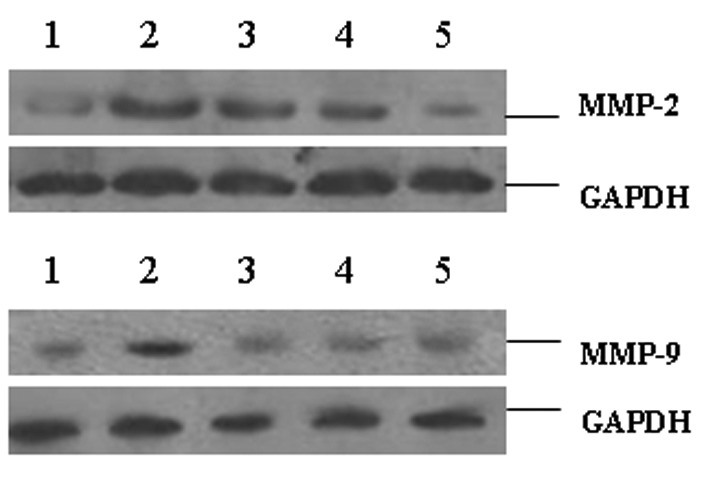
Effect of Xiaochaihu Tang (XCHT) on protein levels of matrix metalloproteinase (MMP)-2 and -9. GAPDH, glyceraldehyde 3-phosphate dehydrogenase. Lane 1, sham surgery; lane 2, model control; lane 3, 7.5 g/kg XCHT group; lane 4, 15 g/kg XCHT group; lane 5: gestrinone.

As shown in Fig. 3 and Table III, the protein levels of MMP-2 and MMP-9 in the ectopic endometria were significantly higher than those in the normal endometria (P<0.001). In the tissues of the XCHT-treated groups, the protein levels of MMP-2 and MMP-9 were significantly decreased compared with those in the model control group (P<0.05 and P<0.001, respectively, for the 7.5 g/kg group; P<0.01 and P<0.001, respectively, for the 15 g/kg group). These results indicate that XCHT reduced the protein levels of MMP-2 and MMP-9.

## Discussion

MMPs are zinc-dependent proteases that preserve the function of disassembling the ECM, thereby participating in the degradation and reconstruction of numerous types of tissues. This function is also closely associated with the invasion and metastasis of cancer cells (7–9). Similar to tumor cells, the ectopic endometrium is able to shed, diffuse, metastasize and invade surrounding tissues and organs. Studies have shown that the expression of MMPs is significantly increased in the ectopic endometrium, indicating that MMPs may participate in the displacement of the endometrium. Among the MMP family, MMP-2 and MMP-9 are closely associated with the formation of EMs (10–12).

Numerous studies have demonstrated that XCHT and its active components exhibit notable therapeutic effect in EMs (4,5). Zheng *et al*(3) observed that XCHT inhibited the growth and angiogenesis of the ectopic endometrium in a rat model, which was most likely associated with the regulation of the immune system. Pan and Zheng (13) suggested that the effect of XCHT on EMs may have been due to the decrease in cyclooxygenase (COX)-2 and P450 levels in the ectopic endometrium and the following reduction of estrogen. Furthermore, Zhang and Wan (14) demonstrated that baicalein exhibited a good therapeutic effect in rats with EMs, with a possible mechanism being through reductions in the levels of tumor necrosis factor (TNF)-α, interleukin (IL)-6 and IL-8, and the inhibition of the expression of intercellular adhesion molecule-1 (ICAM-1) and Bcl-2. Song *et al*(15) revealed that ginsenoside Rg3 inhibited the expression of inhibitor of DNA binding 1 (ID-1) and neuropilin-1 (NRP1) in EMs cells. However, it was unclear whether XCHT was able to reverse the abnormal expression of MMP-2 and MMP-9 in EMs. In the present study, we demonstrated that the mRNA and protein levels of MMP-2 and MMP-9 were significantly higher in the ectopic endometrium than those in the normal endometrium, which further indicated that MMP-2 and MMP-9 were involved in pathogenesis of EMs. We also showed that XCHT was able to significantly downregulate the mRNA and protein expression levels of MMP-2 and MMP-9 in the ectopic endometrium, which suggests that the therapeutic effect of XCHT on EMs may have been due to the inhibition of MMP-2 and MMP-9.

In conclusion, XCHT is able to decrease the expression of MMP-2 and MMP-9 in the ectopic endometrium. The present results may provide a potential theoretical basis for the therapy of EMs.

## Figures and Tables

**Table I:** PCR primers and temperature (Tm).

Gene	Primer sequence	Tm (°C)	Product size (bp)
MMP-2	Sense: 5′-GGCCCTGTCACTCCTGAGAT-3′ Antisense: 5′-GGCATCCAGGTTATCGGGGA-3′	56	326
MMP-9	Sense: 5′-GATGCGTGGAGAGTCGAAAT-3′ Antisense: 5′-CACCAAACTGGATGACGATG-3′	58	273
GAPDH	Sense: 5′-CAGGGCTGCTTTTAACTCTG-3′ Antisense: 5′-GATGATCTTGAGGCTGTTGTC-3′	58	385

PCR, polymerase chain reaction; MMP, matrix metalloproteinase; GAPDH, glyceraldehyde 3-phosphate dehydrogenase.

**Table II:** Effect of XCHT on mRNA levels of MMP-2 and MMP-9.

Group	Dose (g/kg)	MMP-2/GAPDH	MMP-9/GAPDH
Sham surgery	-	0.77±0.24	0.83±0.36
Model control	-	1.24±0.35^a^	1.87±0.33^b^
XCHT	7.5	1.06±0.37^c^	0.89±0.27^e^
XCHT	15	0.72±0.31^d^	0.87±0.36^e^
Gestrinone	0.0005	0.64±0.21^d^	0.99±0.34^e^

a P<0.01,

b P<0.001 vs. the sham surgery group;

c P<0.05,

d P<0.01,

e P<0.001 vs. the model control group.

XCHT, Xiaochaihu Tang; MMP, matrix metalloproteinase; GAPDH, glyceraldehyde 3-phosphate dehydrogenase.

**Table III:** Effect of XCHT on protein levels of MMP-2 and MMP-9.

Group	Dose (g/kg)	MMP-2/GAPDH	MMP-9/GAPDH
Sham surgery	-	0.27±0.06	0.32±0.09
Model control	-	0.87±0.24^a^	0.96±0.24^a^
XCHT	7.5	0.69±0.12^b^	0.41±0.11^d^
XCHT	15	0.54±0.14^c^	0.37±0.09^d^
Gestrinone	0.0005	0.31±0.11^d^	0.33±0.12^d^

a P<0.001 vs. the sham surgery group;

b P<0.05,

c P<0.01,

d P<0.001 vs. the model control group.

XCHT, Xiaochaihu Tang; MMP, matrix metalloproteinase; GAPDH, glyceraldehyde 3-phosphate dehydrogenase.
